# Crystal structure of the plant GABA aminotransferase *At*GABA-T from *Arabidopsis thaliana*

**DOI:** 10.1107/S2053230X26003456

**Published:** 2026-05-01

**Authors:** Naofumi Okoda, Suguru Okuda, Kenta Tsutsumi, Hideaki Itoh, Ken Okamoto, Hiroshi Kawakami, Kaori Sano, Kenji Kobata, Koji Nagata

**Affiliations:** a Department of Applied Biological Chemistry, Graduate School of Agricultural and Life Sciences The University of Tokyo Tokyo113-8657 Japan; b Research Center for Food Safety, Graduate School of Agricultural and Life Sciences The University of Tokyo Tokyo113-8657 Japan; c Department of Chemistry and Biological Science, Faculty of Science Josai University Saitama350-0295 Japan; d Graduate School of Pharmaceutical Sciences Josai University Saitama350-0295 Japan; e Agricultural Bioinformatics Research Unit, Graduate School of Agricultural and Life Sciences The University of Tokyo Tokyo113-8657 Japan; University of Leipzig, Germany

**Keywords:** *Arabidopsis thaliana*, crystal structure, GABA aminotransferases, pyridoxal 5′-phosphate

## Abstract

γ-Aminobutyric acid aminotransferase (GABA-T) is a pyridoxal 5′-phosphate (PLP)-dependent enzyme that catalyzes γ-aminobutyric acid (GABA) degradation in the mitochondrial GABA shunt. Plant GABA-Ts correspond to EC 2.6.1.96 and differ from mammalian and bacterial GABA-Ts (EC 2.6.1.19) in substrate specificity; however, their structural basis has remained unclear. Here, we report the crystal structure of GABA aminotransferase from *Arabidopsis thaliana* (*At*GABA-T) at 2.0 Å resolution. Structural comparison using *Foldseek* indicates that *At*GABA-T belongs to the class III aminotransferase family. Comparison with a class III ω-aminotransferase suggests that Arg423 located in the C-terminal region of *At*GABA-T functions as the primary anchor for the carboxyl group of GABA, together with residues forming a tunnel-shaped substrate-access pathway. In contrast to nonplant GABA aminotransferases, which utilize a conserved N-terminal arginine for substrate recognition, *At*GABA-T employs a distinct C-terminal arginine. These findings provide direct structural evidence for the classification of *At*GABA-T as a class III aminotransferase and reveal a distinct mode of substrate recognition in *At*GABA-T.

## Abstract 2

The crystal structure of *A. thaliana* γ-aminobutyric acid aminotransferase (GABA-T) reveals that plant GABA-T belongs to the class III aminotransferase family and employs a C-terminal arginine residue for γ-aminobutyric acid recognition, in contrast to the N-terminal arginine used by class II enzymes.

## 1. Introduction

γ-Aminobutyric acid (GABA) is a nonproteinogenic amino acid that is widely distributed in plants, animals and microorganisms, where it functions in both metabolism and signaling. In most organisms, GABA is catabolized through the so-called GABA shunt, a metabolic bypass of the tri­carboxylic acid (TCA) cycle that converts GABA to succinate via two enzymatic steps. GABA aminotransferase (GABA-T) catalyzes the first step of this pathway by transferring the amino group of GABA to an α-keto acid acceptor, yielding succinic semialdehyde and the corresponding amino acid. This reaction is dependent on the cofactor pyridoxal 5′-phosphate (PLP), and GABA-Ts belong to the aminotransferase superfamily (Fait *et al.*, 2008 ▸).

Two types of GABA aminotransferase activity have been described based on substrate specificity and enzyme classification. GABA-Ts corresponding to EC 2.6.1.19, which preferentially use α-ketoglutarate as the amino acceptor, are found in mammals, fungi and many bacteria. In contrast, plant GABA-Ts correspond to EC 2.6.1.96 and preferentially use pyruvate and glyoxylate as the amino acceptor (Clark *et al.*, 2009 ▸). In plants, the GABA shunt is closely linked to carbon–nitrogen balance, pH regulation and responses to biotic and abiotic stresses. GABA levels rapidly increase in response to environmental stimuli such as salinity, mechanical damage and pathogen attack, indicating an important role for GABA metabolism in stress adaptation (Renault *et al.*, 2010 ▸).

In *Arabidopsis thaliana*, a mitochondrial GABA aminotransferase encoded by the *POP2* gene (*At*GABA-T) has been genetically and biochemically characterized (Clark *et al.*, 2009 ▸). Loss-of-function mutations in *POP2* result in the accumulation of GABA and altered levels of downstream metabolites, leading to defects in reproductive development and stress responses (Palanivelu *et al.*, 2003 ▸; Renault *et al.*, 2010 ▸). These observations underscore the physiological importance of *At*GABA-T in maintaining GABA homeostasis *in planta*.

Extensive biochemical and structural studies have been conducted on GABA aminotransferases from mammals and microorganisms, revealing conserved catalytic mechanisms of PLP-dependent transamination and identifying key residues involved in cofactor and substrate binding (Koper *et al.*, 2022 ▸). These enzymes are typically classified as class II aminotransferases and utilize a conserved arginine residue located in the N-terminal region to anchor the carboxyl group of GABA. In contrast, plant GABA aminotransferases have been predicted from sequence analyses to belong to the class III aminotransferase family; however, direct structural evidence supporting this classification and elucidating the substrate-recognition mechanism of plant GABA-Ts has remained limited.

To address this gap, we determined the crystal structure of GABA aminotransferase from *A. thaliana* (*At*GABA-T). Structural comparison using the *Foldseek* server (van Kempen *et al.*, 2024 ▸) supports the conclusion that *At*GABA-T belongs to the class III aminotransferase family. Furthermore, comparison with a class III aminotransferase for which a substrate-complex structure has been reported provides insights into the molecular basis of GABA recognition by *At*GABA-T. These structural data reveal a mode of substrate recognition that differs from that of mammalian and bacterial GABA aminotransferases and provide a framework for understanding the unique features of plant GABA metabolism.

## 2. Materials and methods

### 2.1. Protein production

The plasmid construct for *At*GABA-T used in this study was based on that previously described by Sano *et al.* (2023 ▸) (Table 1 ▸). *At*GABA-T contains an N-terminal mitochondrial targeting signal consisting of 36 amino-acid residues; this region was removed for recombinant expression. The truncated *At*GABA-T gene was cloned into the pET-3c vector with a C-terminal 5×His tag and transformed into *Escherichia coli* KRX cells. Transformed cells were grown overnight at 310 K in 10 ml Luria–Bertani (LB) medium supplemented with 50 µg ml^−1^ ampicillin. The overnight culture was used to inoculate 1.0 l LB medium containing 50 µg ml^−1^ ampicillin and was grown at 310 K with shaking until the optical density at 600 nm reached 0.6–1.0. Protein expression was induced by the addition of 1.0 g rhamnose, and the culture was further incubated overnight at 289 K with shaking. The cells were harvested by centrifugation at 4000*g* for 30 min at 277 K and were stored at 193 K until use. The cell pellet was resuspended in 35 ml phosphate-buffered saline (PBS) pH 7.4 on ice and lysed by homogenization. Cell debris was removed by centrifugation at 40 000*g* for 30 min at 277 K, and the resulting supernatant was applied onto an Ni–NTA affinity column (Bio-Rad) pre-equilibrated with PBS at 277 K. After washing with wash buffer consisting of 50 m*M* sodium phosphate buffer pH 8.0, 150 m*M* NaCl, 20–40 m*M* imidazole, the bound protein was eluted with elution buffer consisting of 50 m*M* sodium phosphate buffer pH 8.0, 150 m*M* NaCl, 100 m*M* imidazole at 277 K. PLP was added to the eluted protein fraction to a final concentration of 1 m*M*, followed by incubation on ice for 1 h. The protein sample was then further purified by size-exclusion chromatography using a Superdex 200 Increase 10/300 GL column (Cytiva) equilibrated with SEC buffer consisting of 50 m*M* HEPES–NaOH pH 7.5, 150 m*M* NaCl, 5 m*M* TCEP at 277 K. The purity of *At*GABA-T was confirmed by SDS–PAGE.

### 2.2. Crystallization

Purified *At*GABA-T was concentrated using an Amicon Ultra-15 centrifugal filter unit with a 50 000 nominal molecular-weight limit (NMWL; Millipore). The protein concentration was adjusted to 5.1 mg ml^−1^ as determined by the absorbance at 280 nm. Initial crystallization screening was performed at 293 K using the sitting-drop vapor-diffusion method in 96-well plates (Violamo) with commercially available screening kits (PEG/Ion; Hampton Research). Drops were prepared by mixing 0.5 µl protein solution with 0.5 µl reservoir solution and equilibrated against 50 µl reservoir solution. Small crystals appeared within one week under several conditions, including PEG/Ion conditions A4, A7 and C4. Optimization of the crystallization conditions was carried out using the hanging-drop vapor-diffusion method in 24-well plates (Hampton Research) at 293 K. Drops were prepared by mixing 1.0 µl protein solution with 1.0 µl reservoir solution and were equilibrated against 500 µl reservoir solution. Diffraction-quality crystals were obtained within one week under optimized conditions consisting of 0.2 *M* calcium acetate hydrate pH 7.6, 18%(*w*/*v*) polyethylene glycol 3350. These conditions were derived from the original PEG/Ion condition C4 [0.2 *M* calcium acetate hydrate pH 7.5, 20%(*w*/*v*) polyethylene glycol 3350].

### 2.3. Cryoprotection, X-ray diffraction data collection and processing

*At*GABA-T crystals were cryoprotected using a two-step protocol. Crystals were first soaked briefly in cryoprotectant I consisting of 0.22 *M* calcium acetate hydrate pH 7.6, 19.8%(*w*/*v*) polyethylene glycol 3350, 10%(*v*/*v*) ethylene glycol. The crystals were then transferred to cryoprotectant II consisting of 0.24 *M* calcium acetate hydrate pH 7.6, 21.6%(*w*/*v*) polyethylene glycol 3350, 20%(*v*/*v*) ethylene glycol, followed by flash-cooling in liquid nitrogen.

X-ray diffraction data were collected at 100 K on beamline BL44XU at SPring-8, Hyogo, Japan and on beamline BL-17A at the Photon Factory, Tsukuba, Japan. Diffraction images were indexed, integrated and scaled using *XDS* (Kabsch, 2010 ▸). The crystal structure analysis was performed using software packages included in *CCP*4 (Agirre *et al.*, 2023 ▸) and *Phenix* (Liebschner *et al.*, 2019 ▸). Initial phases were obtained by molecular replacement using *MOLREP* (Vagin & Teplyakov, 1997 ▸) with a structural model predicted by *AlphaFold*3 (Abramson *et al.*, 2024 ▸) as the search template. Model building and refinement were performed using *REFMAC*5 (Murshudov *et al.*, 2011 ▸), *Coot* (Emsley & Cowtan, 2004 ▸) and *phenix.refine* (Afonine *et al.*, 2012 ▸). Polder maps were calculated using *Phenix* (Liebschner *et al.*, 2017 ▸) to validate ligand density. Molecular graphics were produced and structural analyses were carried out using *PyMOL* (Schrödinger).

### 2.4. Comparative sequence analysis

The representative plant POP2 homologs shown in Supplementary Fig. S1 were selected from the supplementary dataset of Koper *et al.* (2024 ▸) to cover phylogenetically diverse plant species. Selected mammalian GABA aminotransferase homologs (Supplementary Fig. S2) and representative bacterial class III aminotransferases identified by *Foldseek* (Supplementary Fig. S3) were included for comparison. Multiple sequence alignments were generated using *Clustal Omega* (Sievers *et al.*, 2011 ▸), and residues corresponding to the N-terminal arginine and *At*GABA-T Arg423 were examined in the aligned sequences.

## 3. Results and discussion

### 3.1. Overall structure

**Figure 1:**
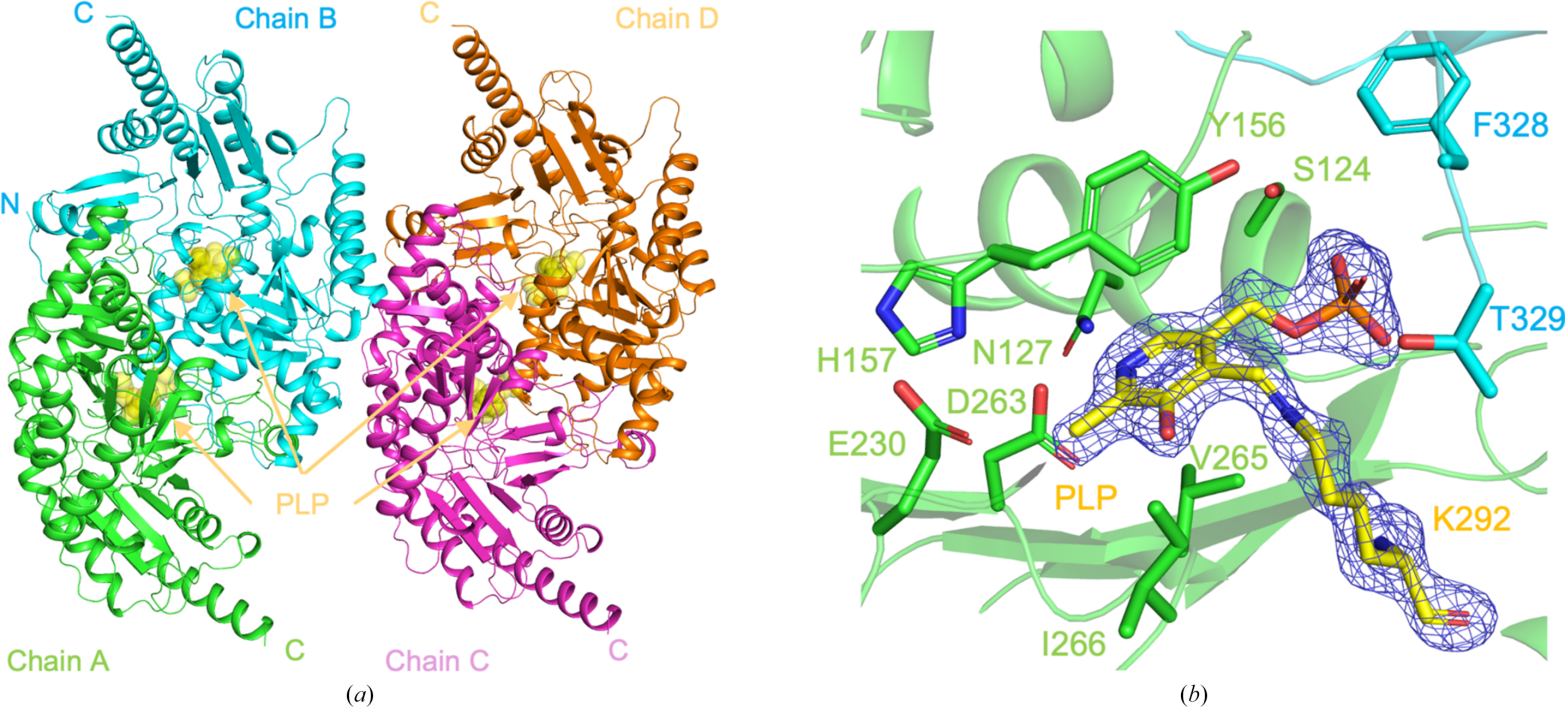
Crystal structure of *A. thaliana* GABA aminotransferase (*At*GABA-T). (*a*) Structural overview of the *At*GABA-T dimers (chains *A*–*B* and *C*–*D*) observed in the crystal lattice. The PLP cofactor is covalently linked to Lys292 in all subunits, forming an internal aldimine. PLP and Lys292 are shown as yellow spheres. (*b*) Close-up view of the active site showing the internal aldimine form of PLP. The main protomer is shown in green and the neighboring protomer is shown in cyan. A polder map contoured at 4.0σ is shown around the PLP cofactor.

The crystal structure of *At*GABA-T was determined at 2.0 Å resolution. Data-collection and refinement statistics are summarized in Table 2 ▸. The asymmetric unit contains two homodimers (chains *A*–*B* and *C*–*D*; Fig. 1 ▸*a*). Structural superposition of chains *B*, *C* and *D* onto chain *A* revealed that the four subunits are highly similar, with root-mean-square deviation (r.m.s.d.) values of 0.143, 0.166 and 0.123 Å, respectively, calculated over all C^α^ atoms.

To identify structural homologs of *At*GABA-T, a structural similarity search of the PDB was performed using the *Foldseek* server. The top structural hits were all annotated as class III aminotransferases (Table 3 ▸), including ω-aminotransferases, putative aminotransferases, pyruvate transaminases and related transaminases from diverse bacterial species. Comparison of these homologs revealed that the residue corresponding to the N-terminal substrate-recognition arginine found in nonplant GABA aminotransferases was replaced by other residues, whereas the position corresponding to *At*GABA-T Arg423 was conserved as an arginine. These results support the assignment of *At*GABA-T to the class III aminotransferase fold and indicate that among the top structural homologs examined here, the substrate-anchoring arginine is located in the C-terminal region.

Clear electron density was observed for the PLP cofactor in all subunits. PLP is covalently linked to Lys292, forming an internal aldimine, and is stabilized by a conserved network of interactions involving residues from both domains (Fig. 1 ▸*b*). These features indicate that *At*GABA-T shares the core structural framework characteristic of PLP-dependent aminotransferases.

### 3.2. Structural basis for GABA recognition

To elucidate the structural basis for GABA recognition by *At*GABA-T, we performed a comparative structural analysis with *Pseudomonas jessenii* ω-aminotransferase (*Pj*AT), a class III aminotransferase for which a substrate-analog complex structure has been reported (PDB entry 6g4e; Palacio *et al.*, 2019 ▸). *Pj*AT utilizes 6-aminohexanoic acid as an amino-group donor, which possesses a longer aliphatic chain than GABA, and thus provides a useful reference for understanding substrate specificity within the class III aminotransferase family.

**Figure 2:**
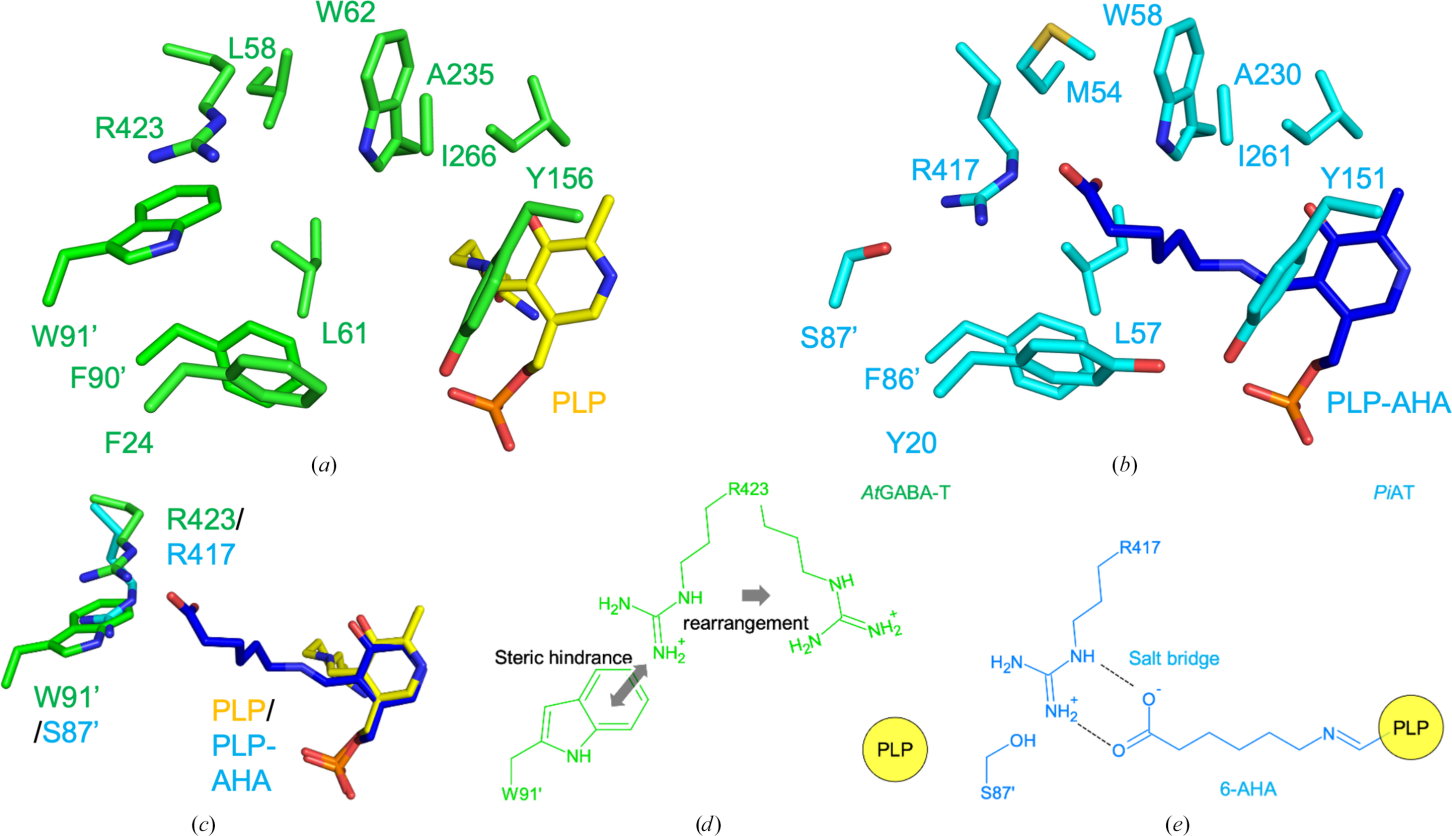
Structural comparison of the substrate-binding sites of *At*GABA-T and *P. jessenii* ω-aminotransferase (*Pj*AT; PDB entry 6g4e). Key residues involved in substrate recognition and the PLP cofactor are shown as stick models. (*a*) Substrate-binding site of *At*GABA-T near the PLP cofactor. Residues forming the substrate-binding pocket are shown, with Arg423 positioned as a putative anchor residue for substrate capture. (*b*) Substrate-binding site of *Pj*AT in complex with the substrate analog 6-aminohexanoic acid (AHA). The external aldimine formed between AHA and PLP is shown. Arg417 of *Pj*AT anchors the carboxylate group of AHA by forming a salt bridge. Dashed lines indicate the salt-bridge and hydrogen-bond interactions between Arg417 and AHA. (*c*) Superposition of the *At*GABA-T and PjAT active sites. Trp91 and Arg423 of *At*GABA-T, Ser87 and Arg417 of *Pj*AT, and the PLP cofactor and PLP–AHA external aldimine are shown as stick models. The superposition highlights that Trp91 of *At*GABA-T occupies a bulkier position than the corresponding serine residue of *Pj*AT, suggesting that this steric difference may influence the local conformational environment of the anchoring arginine near the ligand-binding site. (*d*) Schematic model illustrating a possible rearrangement of the anchor residue in *At*GABA-T [corresponding to (*a*)]. A two-dimensional schematic representation of the *At*GABA-T substrate-binding site illustrates a possible spatial relationship between Arg423 and Trp91 (corresponding to Ser87 in *Pj*AT). This model suggests that accommodation of GABA may require local conformational adjustment of Arg423 to allow salt-bridge formation with the substrate carboxylate. (*e*) Schematic model of the substrate-binding mode in *Pj*AT. A two-dimensional representation of the *Pj*AT active site [corresponding to (*b*)] illustrates the established binding mode, in which Arg417 stably anchors the carboxylate group of AHA and the surrounding residues define the substrate-access channel.

Structural superposition of *At*GABA-T and *Pj*AT resulted in a root-mean-square deviation (r.m.s.d.) of 0.66 Å over all C^α^ atoms, indicating a high degree of structural similarity (Figs. 2 ▸*a* and 2 ▸*b*). In the *Pj*AT–substrate analog complex, the terminal carboxylate group of the substrate is anchored by Arg417 located in the C-terminal region. Arg423 is the corresponding residue of *At*GABA-T and the side chain also points into the substrate-binding pocket, strongly suggesting that this residue functions as the primary anchor for the carboxyl group of GABA in *At*GABA-T.

Despite this positional conservation of the arginine residue, differences are observed in the active-site entrance that are likely to contribute to substrate specificity (Figs. 2 ▸*c*–2 ▸*e*). In *At*GABA-T (Fig. 2 ▸*d*), Trp91′ (where the prime indicates a residue from the partner subunit) is positioned adjacent to Arg423, and its bulky aromatic side chain is expected to restrict the orientation and mobility of Arg423, thereby favoring binding of the shorter substrate GABA. In contrast, in *Pj*AT (Fig. 2 ▸*e*) the corresponding residue, Ser87′, creates a more spacious substrate-binding pocket around Arg417. The additional space may facilitate accommodation of the longer substrate 6-aminohexanoate (AHA). These structural differences suggest a possible mechanism by which substrate-length discrimination is achieved within class III aminotransferases.

In addition to the anchoring arginine, several residues contribute to shaping a tunnel-like pathway leading from the protein surface to the PLP cofactor. In *At*GABA-T, Trp62 is positioned at the entrance to the active site and is likely to function as a gatekeeper residue, forming a hydrophobic environment suitable for the aliphatic chain of GABA. Tyr156 constitutes part of the tunnel wall and further contributes to the hydrophobic character of the substrate-binding pathway. These residues correspond well to Trp58 and Tyr151 in *Pj*AT, respectively, indicating a conserved architecture for substrate access among class III aminotransferases.

Taken together, these structural observations suggest that *At*GABA-T recognizes GABA through anchoring of the substrate carboxyl group by Arg423 in the C-terminal region, combined with size and shape selection mediated by residues lining the substrate-access tunnel. This mode of substrate recognition provides a structural basis for understanding the specificity of plant GABA aminotransferases and sets the stage for comparison with GABA aminotransferases from other enzyme classes.

### 3.3. Structural comparison to bacterial and mammalian aminotransferases that use an arginine in the N-terminal region as an anchor for the substrate carboxylate group

To clarify the molecular basis of GABA recognition by *At*GABA-T, we compared its active-site architecture with those of representative bacterial and mammalian GABA aminotransferases. Structural analyses of canonical GABA aminotransferases from *Corynebacterium glutamicum* (PDB entry 6j2v; Hong & Kim, 2019 ▸) and *Arthrobacter aurescens* (PDB entry 4atq; Bruce *et al.*, 2012 ▸), both classified as EC 2.6.1.19 enzymes, have shown that substrate recognition relies on a conserved arginine residue located in the N-terminal region, which anchors the carboxyl group of GABA.

**Figure 3:**
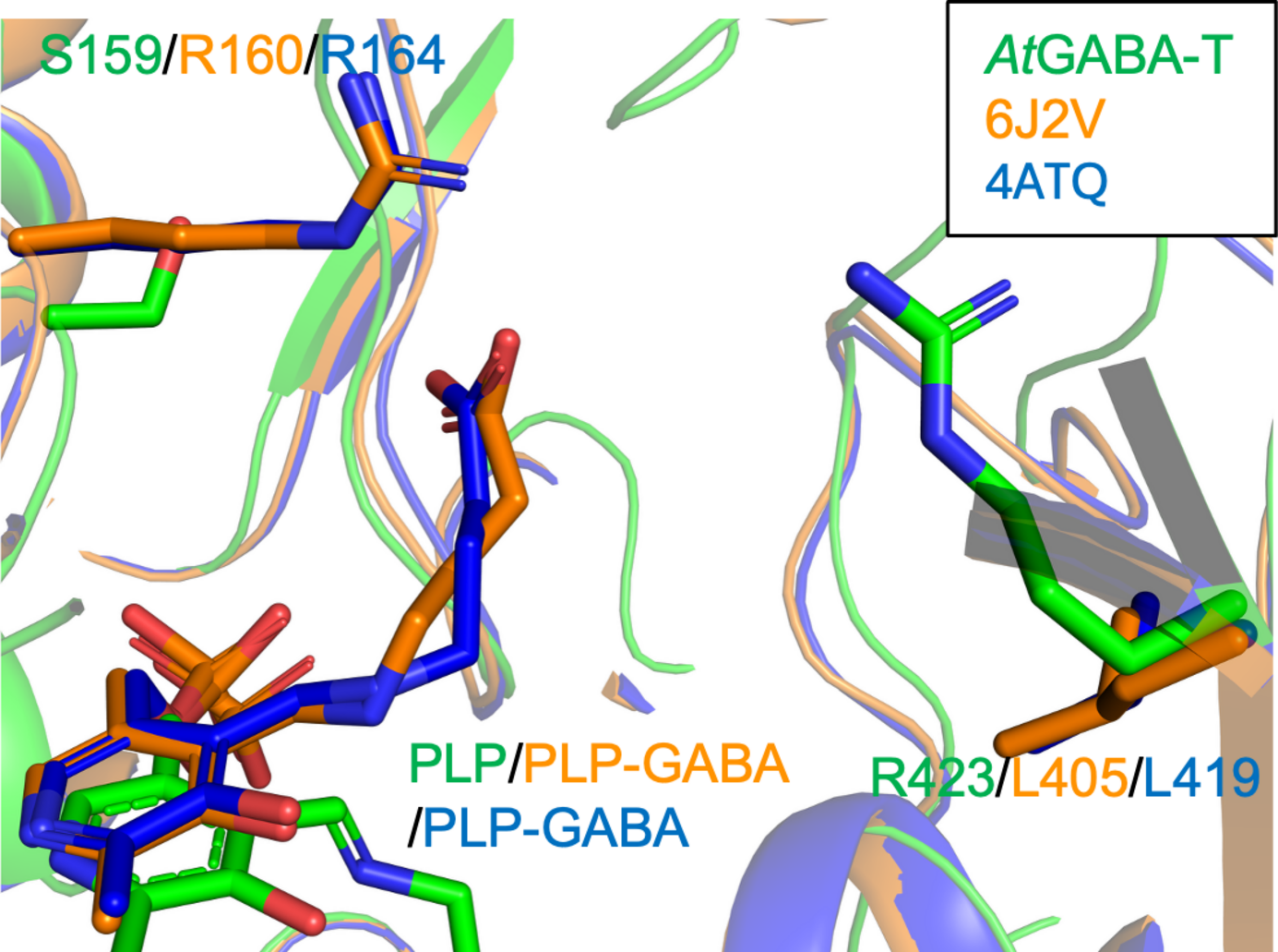
Structural superimposition of the active-site region of *At*GABA-T and bacterial GABA aminotransferase homologs. The active site of *At*GABA-T is superimposed with those of bacterial GABA aminotransferases from *Corynebacterium glutamicum* (PDB entry 6j2v) and *Arthrobacter aurescens* (PDB entry 4atq). Key residues involved in substrate carboxylate recognition, including the N-terminal anchoring arginine in the bacterial enzymes and the corresponding region in *At*GABA-T, are shown as stick models.

In *At*GABA-T, this conserved N-terminal arginine is absent and the corresponding position is occupied by Ser159 (Fig. 3 ▸). Instead, Arg423 located in the C-terminal region is positioned within the active site and is suitably oriented to interact with the substrate carboxyl group. Structural superposition revealed that Arg423 occupies a position distinct from the N-terminal anchoring arginine in bacterial enzymes, indicating a fundamentally different arrangement of charged residues within the substrate-binding pocket.

Detailed comparison of the active-site environments further highlights this difference. In some bacterial GABA aminotransferases, the N-terminal arginine is supported by surrounding residues that stabilize its orientation for substrate capture. In *At*GABA-T, Arg423 is embedded in a different local environment, with neighboring residues shaping a tunnel-like access pathway to the PLP cofactor. This rearrangement results in a redistribution of electrostatic features within the active site, while preserving the overall catalytic framework.

These observations suggest that *At*GABA-T employs a distinct residue arrangement for GABA recognition, in which the primary carboxylate-anchoring role appears to be shifted from the N-terminal to the C-terminal region of the enzyme. Comparative sequence analysis further suggests that this C-terminal arginine arrangement is broadly conserved among the representative plant POP2 homologs examined here, whereas animal GABA aminotransferases more commonly retain the N-terminal arginine (Supplementary Figs. S1 and S2).

### 3.4. Structural classification based on fold-type architecture

The positioning of the substrate-anchoring arginine residue in *At*GABA-T provides a key criterion for its structural classification within the aminotransferase superfamily. Canonical GABA aminotransferases classified as EC 2.6.1.19 are generally assigned to class II aminotransferases and utilize a conserved N-terminal arginine residue to anchor the carboxyl group of GABA.

In *At*GABA-T, this N-terminal arginine is absent and is replaced by Ser159, while Arg423 located in the C-terminal region occupies a position suitable for carboxylate anchoring. This residue arrangement closely mirrors that observed in class III aminotransferases, such as the ω-aminotransferase from *P. jessenii* (PDB entry 6g4e; Palacio *et al.*, 2019 ▸). Consistent with this structural observation, sequence analysis of representative bacterial aminotransferases further supports this distinction: in class II enzymes the N-terminal arginine-equivalent position is conserved, whereas in class III homologs the corresponding position is not conserved and an arginine residue is instead found at the position corresponding to *At*GABA-T Arg423 (Supplementary Fig. S3).

**Figure 4:**
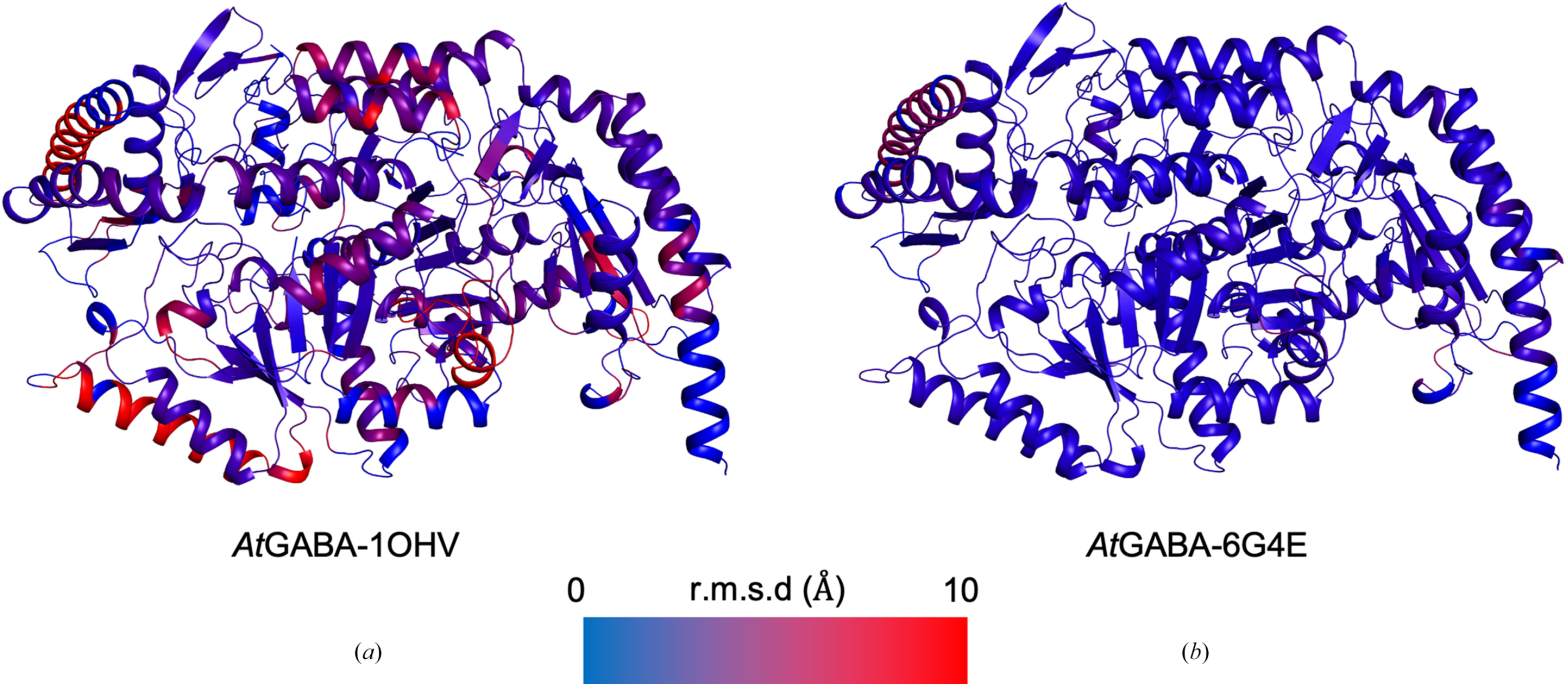
Structural comparison of *At*GABA-T with class II and class III aminotransferases. (*a*) Superposition of *At*GABA-T with *Sus scrofa* GABA aminotransferase (PDB entry 1ohv), a representative class II aminotransferase. (*b*) Superposition of *At*GABA-T with an ω-transaminase (PDB entry 6g4e), a representative class III aminotransferase. In both panels, the query structures are colored according to the C^α^ root-mean-square deviation (r.m.s.d.) values relative to *At*GABA-T. The color gradient ranges from blue (0 Å) to red (10 Å), indicating high and low structural conservation, respectively. The overall fold of *At*GABA-T shows greater structural similarity to the class III ω-transaminase than to the class II enzyme.

Global structural comparisons further support this classification. Structural superposition of *At*GABA-T with mammalian class II GABA aminotransferase from *Sus scrofa* and with a class III ω-aminotransferase indicates that *At*GABA-T shows greater overall structural similarity to the class III enzyme than to the class II enzyme (Fig. 4 ▸). Analysis of C^α^ r.m.s.d. values indicates that the overall fold of *At*GABA-T is more closely conserved with class III aminotransferases than with canonical class II GABA aminotransferases.

Taken together, both the local active-site architecture and the global fold characteristics support the conclusion that *At*GABA-T is best classified as a class III aminotransferase. The C-terminal localization of the substrate-anchoring arginine therefore appears to be a characteristic feature of *At*GABA-T and related plant POP2 homologs, while a similar arrangement is also observed in representative bacterial class III aminotransferases, as supported by the sequence analysis shown in Supplementary Fig. S3.

## 4. Conclusion

In this study, we determined the crystal structure of *A. thaliana* GABA aminotransferase (*At*GABA-T), providing direct structural evidence that *At*GABA-T belongs to the class III aminotransferase family. Structural analyses revealed that *At*GABA-T recognizes GABA through a C-terminal arginine residue, in contrast to the N-terminal arginine employed by mammalian and bacterial GABA aminotransferases. Despite this difference in substrate recognition, *At*GABA-T retains the conserved PLP-dependent fold characteristic of aminotransferases.

Together, these findings provide a structural basis for the distinctive mode of GABA recognition in *At*GABA-T. Comparative analysis further suggests that representative plant POP2 homologs examined here share this class III-type residue arrangement, and that a similar C-terminal arginine arrangement is also conserved in representative bacterial class III aminotransferases identified by *Foldseek*.

## Supplementary Material

PDB reference: GABA aminotransferase from *Arabidopsis thaliana*, 21ft

Supplementary Figures. DOI: 10.1107/S2053230X26003456/no5216sup1.pdf

## Figures and Tables

**Table 1:** Macromolecule-production information

Protein	*At*GABA-T
Expression vector	pET-3c
Expression host	*E. coli* KRX
Complete amino-acid sequence of the construct produced	MTTEAAPDKKNTVGSKGHDMLAPFTAGWQSADLDPLVIAKSEGSYVYDDTGKKYLDSLAGLWCTALGGNEPRLVSAAVEQLNTLPFYHSFWNRTTKPSLDLAKVLLEMFTANKMAKAFFTSGGSDANDTQVKLVWYYNNALGRPEKKKFIARKKSYHGSTLISASLSGLPPLHQNFDLPAPFVLHTDCPHYWRFHLPGETEEEFSTRLAKNLEDLIIKEGPETIGAFIAEPVMGAGGVIPPPATYFEKVQAVVKKYDILFIADEVICAFGRLGTMFGCDKYNIKPDLVTLAKALSSAYMPIGAILMSQEVADVINSHSSKLGVFSHGFTYSGHPVSCAVAIEALKIYKERNIPEYVAKVAPRFQDGVKAFASGSPIIGETRGTGLILGTEFVDNKSPNEPFPPEWGVGAFFGAECQKHGMLVRVAGDGILMSPPLIISPEEIDELISIYGKALKATEEKVKELKAQHKKHHHHH

**Table 2:** Crystallographic data and refinement statistics Values in parentheses are for the highest resolution shell.

Data-collection statistics	
Diffraction source	BL44XU, SPring-8
Wavelength (Å)	0.89995
Temperature (K)	100
Detector	EIGER 16M
Space group	*P*1
Camera distance (mm)	320
Rotation range per image	0.1
Total rotation range (°)	360
Exposure time per image (s)	0.1
*a*, *b*, *c* (Å)	62.178, 78.268, 93.216
α, β, γ (°)	96.763, 88.881, 106.113
Resolution (Å)	50.0–2.00 (2.12–2.00)
Total No. of reflections	392712 (66602)
No. of unique reflections	110288 (17782)
Completeness (%)	96.8 (96.5)
Average *I*/σ(*I*)	10.79 (6.32)
*R*_merge_	0.117 (0.400)
CC_1/2_ (%)	99.1 (92.2)
Refinement statistics	
No. of reflections used	110263
*R*_work_ (%)	15.31
*R*_free_ (%)	19.84
R.m.s.d., bond distances (Å)	0.007
R.m.s.d., bond angles (°)	0.865
No. of atoms	
Protein	14000
Ligand	96
Solvent	1150
*B* factors (Å^2^)	
Protein (*At*GABA-T)	21.95
Ligand (PLP)	19.39
Solvent (water)	28.79
Ramachandran plot	
Residues in favored regions (%)	97.11
Residues in allowed regions (%)	2.67
Residues in outlier regions (%)	0.22

**Table 3:** Structural homologs of *At*GABA-T identified by a *Foldseek* search against the PDB The table lists the top structural hits, their protein annotations, source organisms, aminotransferase class, the residue present at the position corresponding to the N-terminal substrate-recognition arginine of nonplant GABA aminotransferases and the residue at the position equivalent to *At*GABA-T Arg423.

PDB code	Protein name	Organism	Class	Residue at equivalent position to N-terminal Arg	Residue at equivalent position to Arg423	References
21ft	GABA aminotransferase	*Arabidopsis thaliana*	III	Ser	Arg423	This study
6g4e	ω-Aminotransferase	*Pseudomonas jessenii*	III	Ile	Arg417	Palacio *et al.* (2019 ▸)
5ghf	ω-Aminotransferase	*Brucella anthropi*	III	Val	Arg417	Han *et al.* (2017 ▸)
4grx	ω-Aminotransferase	*Paracoccus denitrificans*	III	Val	Arg415	Rausch *et al.* (2013 ▸)
3gju	Putative aminotransferase	*Mesorhizobium japonicum*	III	Ser	Arg420	Joint Center for Structural Genomics (unpublished work)
3fcr	Putative aminotransferase	*Ruegeria* sp. TM1040	III	Ser	Arg420	Joint Center for Structural Genomics (unpublished work)
6io1	(*S*)-Enantioselective ω-transaminase	*Thermomicrobium roseum*	III	Ile	Arg416	Kwon *et al.* (2019 ▸)
3nui	Pyruvate transaminase	*Vibrio fluvialis*	III	Val	Arg415	H. H. Park & T. Jang (unpublished work)
6s54	ω-Aminotransferase	*Pseudomonas fluorescens*	III	Leu	Arg424	Roura Padrosa *et al.* (2019 ▸)
6gwi	Transaminase	*Halomonas elongata*	III	Ser	Arg412	Planchestainer *et al.* (2019 ▸)
5lh9	ω-Aminotransferase	*Pseudomonas* sp.	III	Val	Arg400	Börner *et al.* (2017 ▸)
