# Supplementary material for: Crystal structure of the plant GABA aminotransferase AtGABA-T from Arabidopsis thaliana
Source: Acta Crystallogr F Struct Biol Commun. 2026 May 1;82(Pt 6):208–15. doi: 10.1107/S2053230X26003456 (PMC13224807; doi:10.1107/S2053230X26003456)
Supplement: Supplementary file 1 [file f-82-00208-sup1.pdf]

STRUCTURAL BIOLOGY
COMMUNICATIONS

Volume 82 (2026)

Supporting information for article:

Crystal structure of plant GABA aminotransferase *At*GABA-T from *Arabidopsis thaliana*

Naofumi Okoda, Suguru Okuda, Kenta Tsutsumi, Hideaki Itoh, Ken Okamoto, Hiroshi Kawakami, Kaori Sano, Kenji Kobata and Koji Nagata

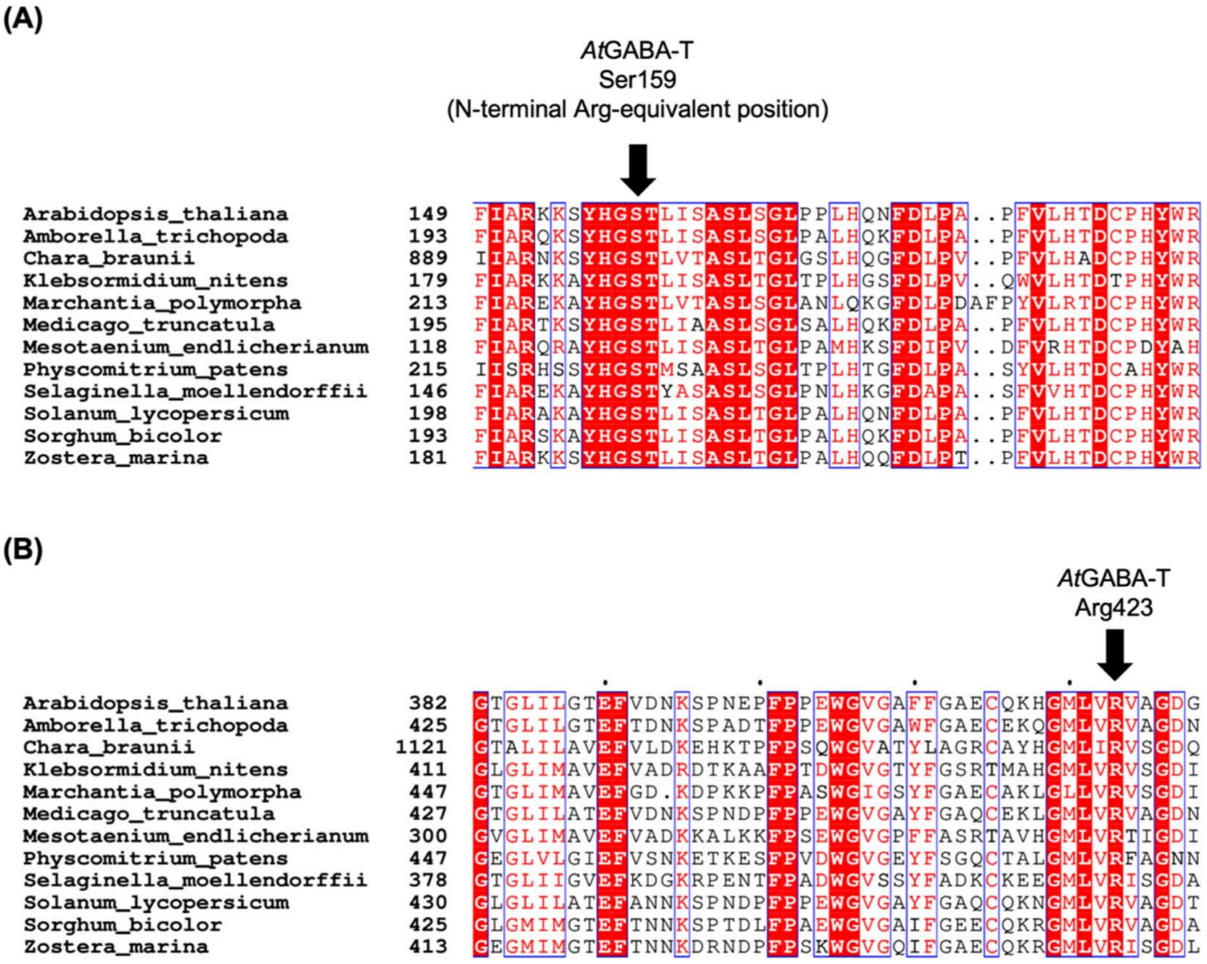

Figure S1 Multiple sequence alignment of representative plant POP2 homologs. **(A)** Sequence segment surrounding the position corresponding to the N-terminal substrate-anchoring arginine of canonical GABA aminotransferases. The arrow indicates *At*GABA-T Ser159, which corresponds to the N-terminal arginine-equivalent position. **(B)** Sequence segment surrounding the position corresponding to *At*GABA-T Arg423. The arrow indicates *At*GABA-T Arg423. Among the representative plant POP2 homologs analyzed here, the N-terminal arginine-equivalent position is not conserved as arginine. In contrast, the Arg423-equivalent position is conserved as arginine in most sequences analyzed.

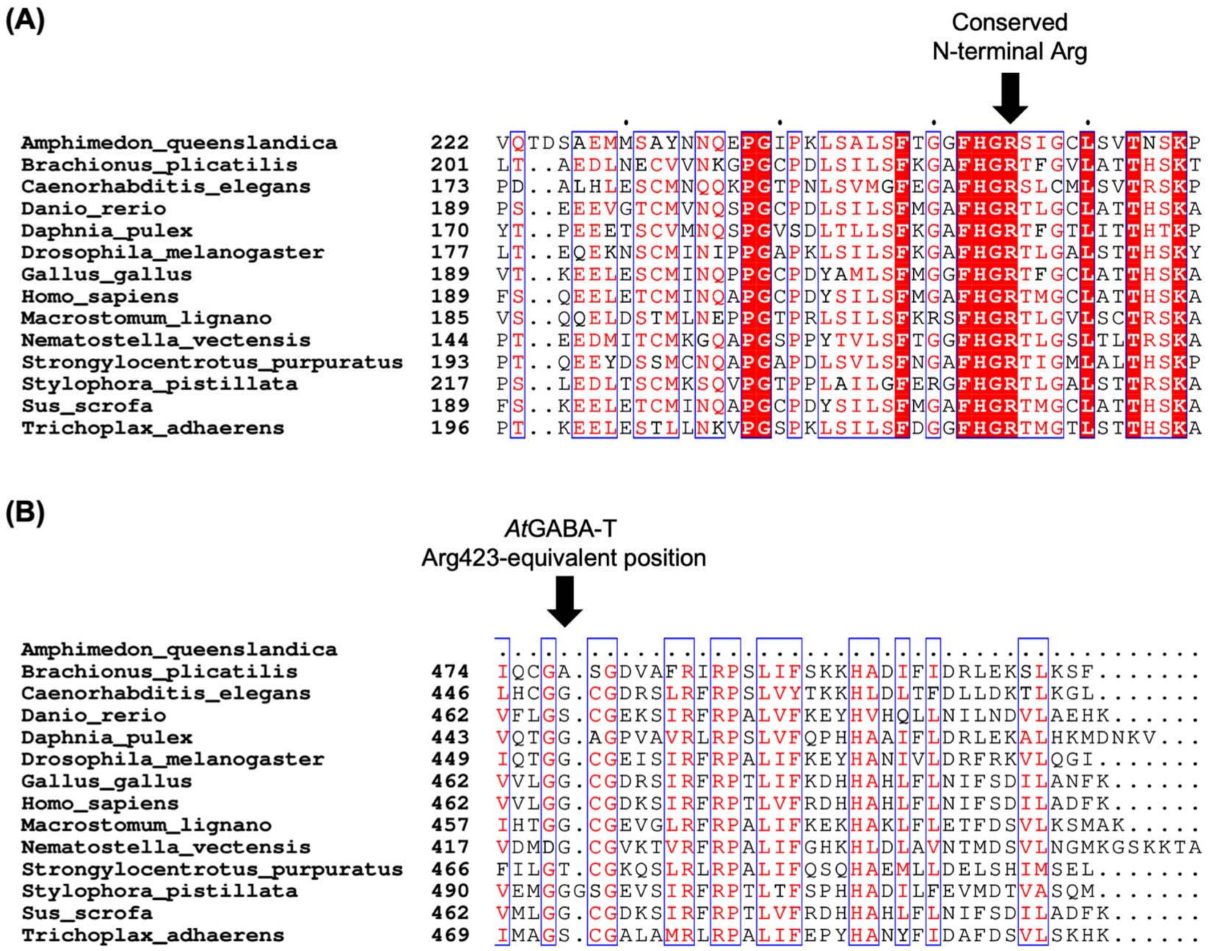

Figure S2 Multiple sequence alignment of representative animal GABA aminotransferase homologs. **(A)** Sequence segment surrounding the position corresponding to the N-terminal substrate-anchoring arginine of canonical GABA aminotransferases. The arrow indicates the conserved N-terminal arginine. **(B)** Sequence segment surrounding the position corresponding to *At*GABA-T Arg423. The arrow indicates the position corresponding to *At*GABA-T Arg423. Among the representative animal GABA aminotransferase homologs analyzed here, the N-terminal arginine-equivalent position is conserved as arginine in most sequences. However, this residue is not retained in *Crassostrea gigas*. In contrast, the Arg423-equivalent position does not show conservation of arginine across the sequences analyzed.

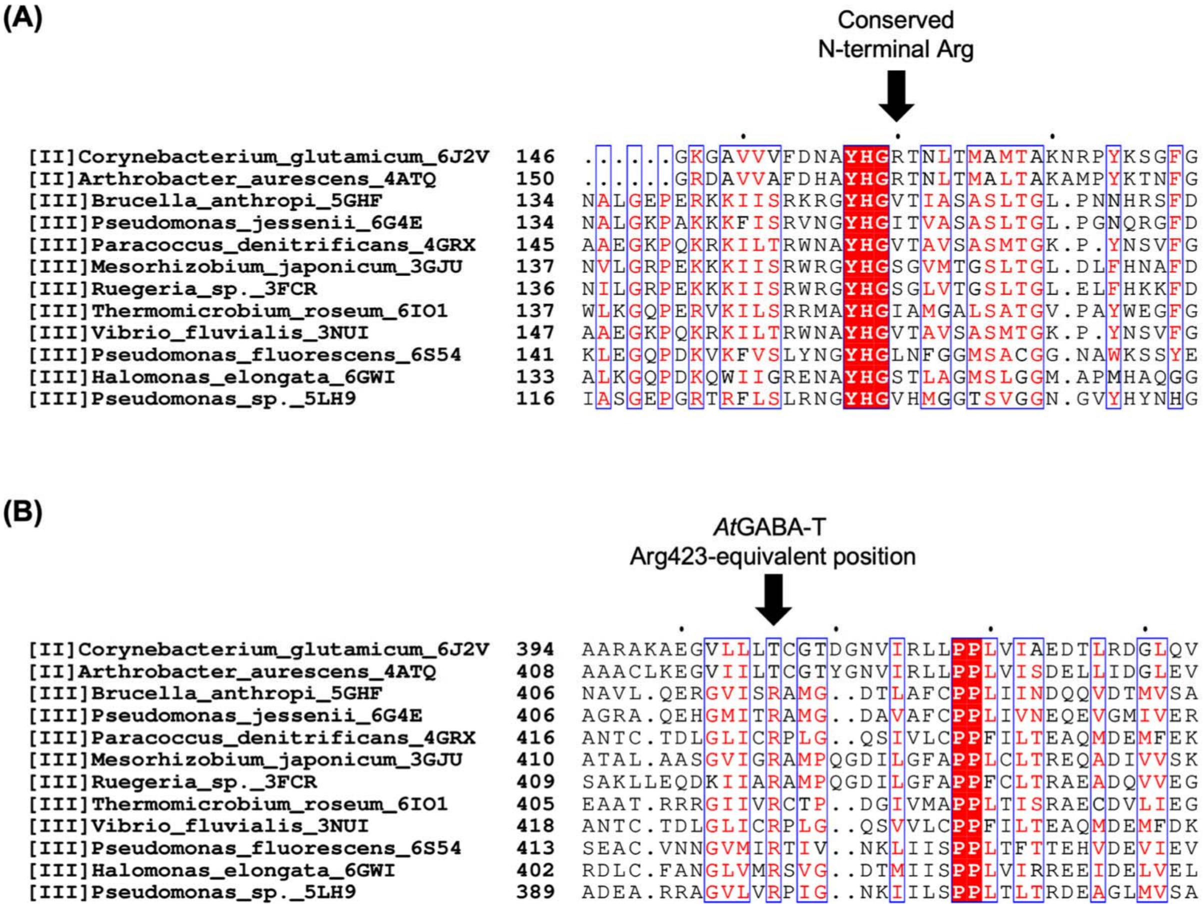

Figure S3 Multiple sequence alignment highlighting distinct positioning of substrate-anchoring arginine residues in bacterial class II and class III aminotransferases. **(A)** Sequence segment surrounding the position corresponding to the N-terminal substrate-anchoring arginine of bacterial class II aminotransferases. **(B)** Sequence segment surrounding the position corresponding to *At*GABA-T Arg423. The first two sequences represent bacterial GABA aminotransferases belonging to class II, whereas sequences 3–12 represent bacterial class III aminotransferase homologs identified by Foldseek. In the class II enzymes, the N-terminal arginine-equivalent position is conserved as arginine, whereas the Arg423-equivalent position does not show conservation of arginine. In contrast, in the class III homologs, the N-terminal arginine-equivalent position is not conserved as arginine, whereas the Arg423-equivalent position is conserved as arginine in most sequences analyzed.
